# Associations Between Virtual Prenatal Care Utilization, Social Determinants of Health, and Stressful Life Events: A Multistate Analysis of U.S. Women, 2020–2021

**DOI:** 10.1007/s10995-026-04289-6

**Published:** 2026-08-06

**Authors:** Don E. Willis, Pearl A. McElfish, Clare C. Brown, Jennifer A. Andersen, George C. Pro, James P. Selig, Cari A. Bogulski

**Affiliations:** 1 School of Human Environmental Sciences, Dale Bumpers College of Agricultural, Food and Life Sciences, University of Arkansas, 118 Home Economics Building, Fayetteville, AR 72701 USA; 2 College of Medicine, University of Arkansas for Medical Sciences, Springdale, AR USA; 3 Institute for Community Health Innovation, University of Arkansas for Medical Sciences Northwest, Springdale, AR USA; 4 Fay W. Boozman College of Public Health, University of Arkansas for Medical Sciences, Little Rock, AR USA; 5 Fay W. Boozman College of Public Health, University of Arkansas for Medical Sciences Northwest, Springdale, AR USA; 6 College of Medicine, University of Arkansas for Medical Sciences Northwest, Fayetteville, AR USA

**Keywords:** Prenatal care, Social determinants, Stress, Virtual care

## Abstract

**Objectives:**

To examine associations between virtual prenatal care utilization, social determinants of health, and stressful life events.

**Methods:**

We analyzed cross-sectional pooled survey data (*N* = 11,966) from phase 8 of the Pregnancy Risk Assessment Monitoring System for women who gave birth in 2020 or 2021 and received prenatal care.

**Results:**

A third (35%) of women utilized virtual rather than in-person-only prenatal care. Adjusted odds of receiving any virtual prenatal care were *greater* for women who identified as Asian (aOR = 1.23; 95% CI[1.01, 1.51]) or Hispanic (aOR = 1.61; 95% CI[1.37, 1.88]), and for those who experienced homelessness (aOR = 1.90; 95% CI[1.27, 2.84]) or job loss (aOR = 1.25; 95% CI[1.04, 1.51]). Adjusted odds were *lower* for women who did not have a bachelor’s degree, had previously given birth, had no insurance pre-pregnancy (aOR = 0.69; 95% CI[0.56, 0.85]), were rural residents (aOR = 0.51; 95% CI[0.41, 0.63]), had been exposed to incarceration (aOR = 0.49; 95% CI[0.30, 0.79]), and had given birth in 2021 (aOR = 0.77; 95% CI[0.69, 0.86]).

**Conclusions:**

Social determinants of health and stressful life events are not uniformly associated with greater or lesser odds of utilizing virtual prenatal care.

## Significance

What is already known on this subject?

What this study adds?

In-person prenatal care utilization is disparate across populations, and its associations with social determinants of health and stressful life events has been well documented. Virtual prenatal care became more common during the COVID-19 pandemic, yet its associations with social determinants of health and stressful life events remain unknown.

This study examined associations between virtual prenatal care utilization and social determinants of health (i.e., race/ethnicity, education, insured status, and rural residence) and stressful life events (e.g., incarceration exposure).

## Introduction

Prenatal care plays a preventive health care role by providing opportunities to perform early screening and treatment for risk factors that might negatively affect maternal and child health. Prenatal care utilization is associated with reduced maternal mortality, pregnancy complications, postpartum depressive/anxiety disorders (Heaman et al., 2019; Yan, 2017), and reductions in preterm birth, low birth weight, and infant mortality (Thurston et al., 2021). Inadequate prenatal care utilization is associated with increased risks for negative health outcomes and behaviors, including insufficient gestational weight gain, prenatal smoking, substance use (Simmons & Austin, 2022), premature rupture of membranes, precipitous labor (Yan, 2017), and maternal mortality (Nelson et al., 2018).

Prenatal care utilization varies across social determinants of health (SDoH) (Blakeney et al., 2019; Slaughter-Acey et al., 2013), or the “social conditions in which people are born, grow, live, work, and age” (Solar & Irwin, 2010). SDoH include “structural stratifiers” (e.g., education) which may impact health by shaping people’s resources, relative social position, as well as “intermediary determinants” such as exposure to stressful life events (SLEs), and access to care (Solar & Irwin, 2010). SDoH such as low education, rurality, minoritized race/ethnicity, low socioeconomic status, and uninsured status or Medicaid enrollment are associated with reduced prenatal care utilization and increased barriers to accessing it (Blakeney et al., 2019; Braveman et al., 2000; Gadson et al., 2017; Radke et al., 2023; Testa & Jackson, 2020).

Prenatal care utilization also varies across stressful life events (SLEs; e.g., incarceration exposure, intimate partner violence) (Testa et al., 2023; Testa & Jackson, 2020). Exposure to SLEs is often influenced by upstream SDoH and structural stratifiers rooted within the broader social and political context (Cohen et al., 2019; Mezuk et al., 2010; Solar & Irwin, 2010). For example, exposure to incarceration is shaped by the intersecting social determinants of education, place/rurality, race/ethnicity, and socioeconomic status and has been found to be associated with barriers to prenatal care (Dumont et al., 2014; Testa et al., 2020). Similar to SDoH, SLEs are often discussed in terms of their direct effect on health outcomes or as a mechanism linking SDoH and health; however, SLEs may also impact health indirectly through health behaviors and health care access (e.g., prenatal care utilization) (Testa & Jackson, 2020). For this reason, research examining relationships between SDoH, SLEs, and prenatal care utilization is essential for understanding the full breadth of pathways through which social conditions, and positions within them, may impact maternal health.

Virtual prenatal care has potential to overcome some disparities observed for in-person prenatal care and, in turn, improve maternal and child health outcomes. Virtual prenatal care may include routine provider visits, remote monitoring, specialist consultations, and genetic counseling. Although it had not been widely adopted prior to 2020, the COVID-19 public health emergency (PHE) sparked substantial increases in the utilization of virtual prenatal care. Virtual prenatal care utilization rose sharply early in the pandemic, declined after late 2020, and remained well above pre-pandemic levels into late 2021 (Acharya et al., 2023). Studies spanning longer periods in 2020 found utilization rates of 33%, 36%, and 37%, indicating around 1 in 3 pregnant women utilized virtual care during this time (Gao et al., 2021; Gourevitch et al., 2023; Lee & Manalew, 2023). Despite the increase in virtual prenatal care utilization and its potential to reduce disparities in care, few studies have examined its relationship with SDoH or SLEs.

Studies examining disparate utilization of virtual prenatal care have produced mixed results. A single-site study conducted between March and July of 2020 found minoritized racial groups were *less* likely to utilize virtual prenatal care compared to white patients (Gao et al., 2021). However, two multi-site studies of births in 2020 and 2021 found some minoritized racial groups (e.g., Hispanic, Asian and Pacific Islanders, and Indigenous groups) were *more* likely than white patients to utilize virtual prenatal care (Gourevitch et al., 2023; Lee & Manalew, 2023). Further, while the single-site study found lower odds of utilization among Medicaid patients than those who were commercially insured (Gao et al., 2021), a national study reported Medicaid patients were more likely to utilize virtual prenatal care but noted their sample consisted almost entirely of commercially insured patients (Acharya et al., 2023). Two multi-state representative studies found no statistically significant differences between those with Medicaid and those with commercial insurance after adjusting for covariates (Gourevitch et al., 2023; Lee & Manalew, 2023). Some studies have found higher odds of virtual prenatal care utilization for rural residents than urban residents (Gao et al., 2021; Gourevitch et al., 2023), but others have found lower odds for rural residents (Hung et al., 2024).

The present study advances the existing literature by utilizing the World Health Organization’s (WHO) Commission on Social Determinants of Health (CSDH) framework to inform the analytical approach and interpretation of findings (Solar & Irwin, 2010). Following the CSDH framework, we examined associations between virtual care and SDoH or proxy indicators of structural stratifiers (e.g., race/ethnicity, education, pre-pregnancy insured status, and rural residence) as well as SLEs or intermediary determinants (Solar & Irwin, 2010). Structural stratifiers intersect to shape socioeconomic positions within hierarchies, and are rooted in institutions and processes further upstream in the social and political context (Solar & Irwin, 2010). Although the CSDH framework makes an important distinction of causal priority by identifying structural and intermediary determinants, it conceptualizes both as social determinants of health (Solar & Irwin, 2010).

The CSDH framework also specifies the health system as a key pathway through which SDoH—structural stratifiers and intermediary determinants—impact health inequities (Solar & Irwin, 2010). The framework specifically notes the issue of access within health systems as critical to how SDoH impact health inequities (Solar & Irwin, 2010, p. 39), further highlighting the importance of examining associations SDoH and virtual prenatal care utilization. Some of the same SDoH predictors we examined in this study have been documented as significantly associated with *in-person* prenatal care utilization (Gourevitch et al., 2023; Testa & Jackson, 2020). However, the relationship between *virtual* prenatal care and SLEs remains largely unknown. We are aware of no other studies that examined associations between virtual prenatal care utilization and SLEs.

Virtual prenatal care offers potential solutions to addressing barriers to prenatal care utilization; (Braveman et al., 2000) however, investigations of the relationship between SDoH, SLEs, and virtual prenatal care utilization remain limited. Given barriers such as technological issues, a lack of private space, and no available virtual appointments, equitable access to prenatal care is not ensured by the innovation of virtual delivery (Lee & Manalew, 2023). The purpose of this study is to fill this gap in the literature by examining whether structural stratifiers (i.e., race/ethnicity, education, pre-pregnancy insured status, and rural residence) and SLEs are associated with virtual prenatal care (alone or in combination with in-person care) utilization.

## Methods

We analyzed cross-sectional pooled survey data from phase 8 of the Pregnancy Risk Assessment Monitoring System (PRAMS) Automated Research File (ARF) for births in 2020 or 2021. PRAMS is a population-based surveillance system administered by health departments in partnership with the Centers for Disease Control and Prevention (CDC). PRAMS surveillance sampling frames are estimated to cover approximately 83% of all United States (US) births (Shulman et al., 2018). During the COVID-19 PHE, several questionnaire supplements were administered to gain additional understanding regarding issues specific to COVID-19. Item nonresponse rates range between 1 and 2% for most items (Shulman et al., 2018).

**Fig. 1:**
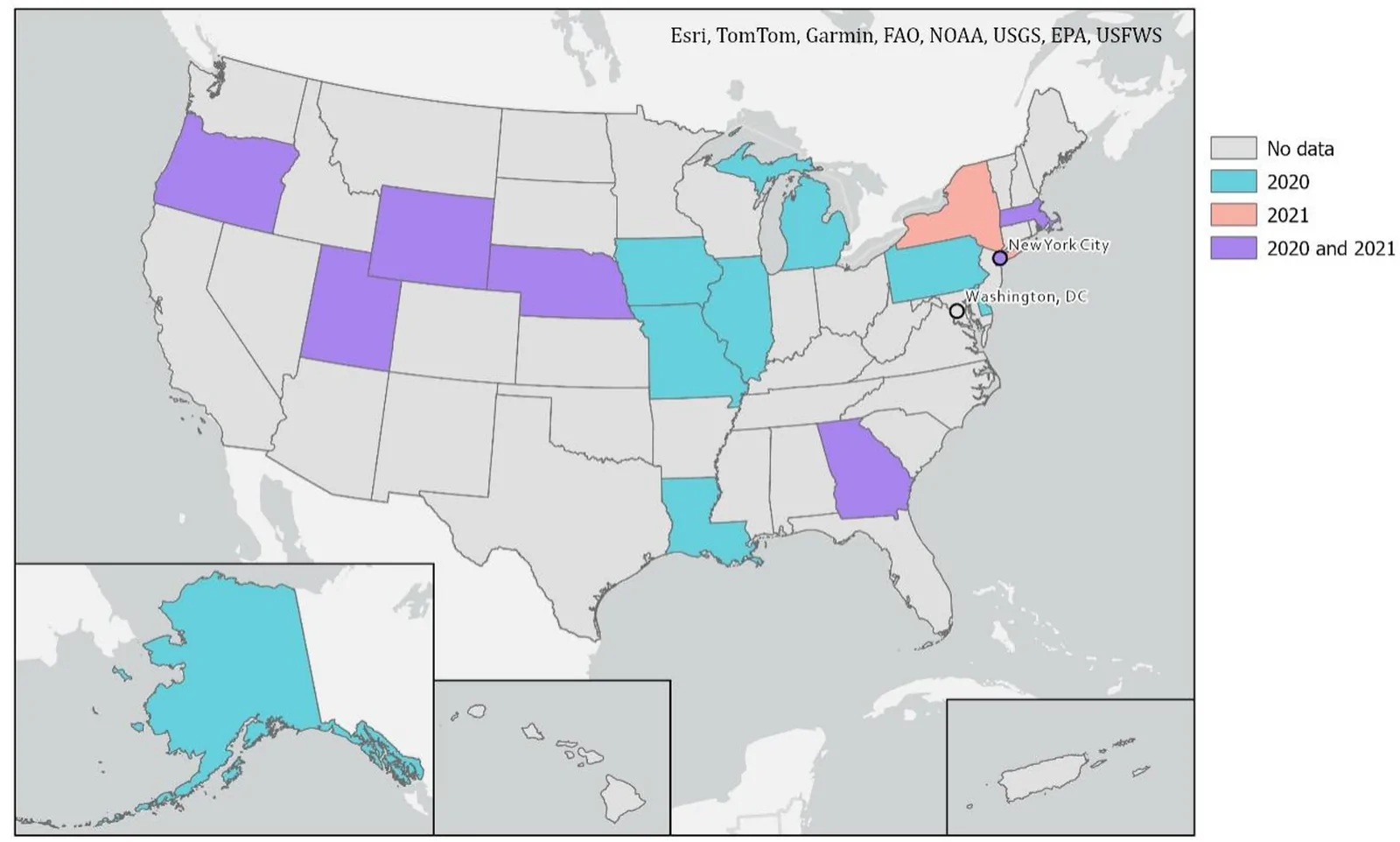
Map indicates sites that assessed *both* virtual prenatal care and SLEs during the 2020 and/or 2021 PRAMS phase 8 distribution (sites *n*=15)

Our analytical sample (*N* = 11,966) includes women who gave birth in 2020 and 2021, received some type of prenatal care during their pregnancy, and had complete data for our variables of interest. Women who reported no prenatal care and were excluded from our analyses made up less than 1% of the sample for 2020 and 2021. Figure 1 displays the geographic coverage of our analytical sample, which includes PRAMS data collection sites that administered *both* the Maternal COVID-19 Experiences supplemental questionnaire (which contains the virtual prenatal care measure) and the SLEs measures from the standard questionnaire in 2020, 2021, or both years. Our analytical sample covers a total of 15 sites across the US, including 14 US states (DE, GA, IA, IL, LA, MA, MI, MO, NE, NY, OR, PA, UT, WY) and New York City. The median response rate for the states and years included in our analytical sample was 57%, with a range of 50–66%. The University of Arkansas for Medical Sciences Institutional Review Board reviewed this study and determined it was not human subjects research due to the use of third-party data without information allowing reidentification.

### Measures

#### Virtual Prenatal Care

Receipt of virtual prenatal care was assessed using the Maternal COVID-19 Experiences questionnaire supplement, which was administered in 2020 and 2021. Respondents were asked, “During the COVID-19 pandemic, which types of prenatal care appointments did you attend?” Response options included, “In-person appointments only,” “virtual appointments (video or telephone) only,” “both, in-person and virtual appointments,” and “I did not have prenatal care.” We analyzed this as a dichotomous variable (any virtual = 1; in-person only = 0) among only those who reported having some type of prenatal care during this time.

#### Age & Pregnancy Characteristics

*Age* was measured in years, calculated from the mother’s date of birth. *Plurality* (single verse multiple) and *previous live births* (0, 1, 2, 3+) were determined from birth certificate variables. *Year of infant birth* (2020 or 2021) was determined from birth certificate data.

#### Social Determinants of Health

We included several measures of SDoH, including race/ethnicity, marital status, educational attainment, pre-pregnancy insured status, and rural residence. *Race/ethnicity* was determined using the ethnicity item (respondents were asked to identify if they were Hispanic, Spanish, or Latina) and the race item (e.g., American Indian or Alaska Native, Asian, Black or African American, Native Hawaiian or Pacific Islander, white, and other race). Aggregation resulted in five mutually exclusive racial/ethnic categorizations: white, non-Hispanic Asian (hereafter Asian), Black, non-Hispanic other/multiracial (hereafter, other/multiracial), and Hispanic. *Marital status* was indicated on birth certificates as either married or other. *Educational attainment* was indicated on birth certificates as less than 8th grade, 9th−12th grade with no diploma, high school graduate/GED, some college with no degree, associate degree, bachelor’s degree, master’s degree, or doctoral or professional degree. Due to low frequencies, we combined all groups with no high school diploma, as well as those with some college/no degree and associate degrees, resulting in four mutually exclusive educational attainment categories. *Insured status* was determined from responses to the question, “During the *month before* you got pregnant with your new baby, what kind of health insurance did you have?” We combined all responses indicating any kind of insurance, creating a dichotomous indicator for women with pre-pregnancy insurance versus those who were not insured. The PRAMS ARF file contains a pre-built indicator of *rural residence* based on the National Center for Health Statistics (NCHS) Urban-Rural Classification Scheme for Counties (Ingram & Franco, 2014).

#### Stressful Life Events

A key predictor variable of interest was exposure to SLEs in the past 12 months. SLEs are assessed using a set of items asking about 14 events respondents may or may not have experienced within the past 12 months (listed in Table 1). Given prior findings that specific SLEs are particularly important predictors of in-person prenatal care or barriers to care (Testa et al., 2020, 2023; Testa & Jackson, 2020) we opted to analyze SLEs as separate items.

#### Data Analysis

We used the Stata/SE software (version 15.1, StataCorp, College Station, TX, 2017) *svy* command to account for the complex survey design (Shulman et al., 2018). We tested for differences in receipt of virtual prenatal care by weighted and unweighted sociodemographic factors, pregnancy history, SDoH, and SLEs using second-order Rao-Scott chi-square tests. Weighted multivariable logistic regression was utilized to examine adjusted odds ratios for receiving any virtual prenatal care. To aid in interpretation, we also computed model-based adjusted predicted probabilities of receiving any virtual prenatal care for each predictor. Statistical significance was determined at an α level of 0.05 for all analyses.

## Results

In Table 1, we present characteristics of US women who gave birth in 2020–2021, received some type of prenatal care during their pregnancy, and had complete data for variables in our analysis (*N* = 11,966). Just over a third (35%) of women who gave birth during this period received only virtual prenatal care or in combination with in-person care, with statistically lower proportions of any virtual prenatal care observed among women who identified their race as Black or other/multiracial, had more previous live births, were not married, had less education, lacked pre-pregnancy insurance, lived in a rural residence, or gave birth in 2021. A lower proportion of any virtual care was observed for women experiencing SLEs, such as problems paying rent/mortgage/bills (30.1% vs. 35.2%; *p*=.006) and or exposure to incarceration (17.3% vs. 34.8%; *p*<.001) either directly (they went to jail) or indirectly (spouse or partner went to jail).

**Table 1:** Characteristics of US women who gave birth in 2020−2021 (*N*=11,966)

	Analytical sample			Any virtual PNC	Sig.
			Weighted	Weighted	Rao-Scott X^2^
	Freq.	Col. %	Col. %	Row %	*p*
Prenatal care type					
In-person prenatal care only	7,613	63.62	65.42	–	–
Any virtual prenatal care	4,353	36.38	34.58	–	–
Age (mean)	11,966	30.01	29.87	30.67	–
Race/Ethnicity					
NH White	4,682	39.13	57.61	33.59	**<0.001**
NH Asian	1,427	11.93	6.37	43.34	
NH Black	1,907	15.94	14.12	29.76	
NH other/multiracial	1,243	10.39	3.08	29.59	
Hispanic	2,707	22.62	18.83	39.07	
Plurality					
Singleton	11,602	96.96	98.51	33.08	0.717
Multiples	364	3.04	1.49	34.60	
Previous live births					
0	5,018	41.94	41.28	38.10	**<0.001**
1	3,679	30.75	31.74	34.34	
2	1,812	15.14	16.19	30.32	
3+	1,457	12.18	10.79	28.21	
Marital status					
Not married	4295	35.89	36.03	30.99	**<0.001**
Married	7671	64.11	63.97	36.60	
Education					
No high school diploma	1,259	10.52	9.68	29.96	**<0.001**
High school graduate	2,703	22.59	24.04	28.26	
Some college, no degree/assoc. degree	3,158	26.39	24.73	30.68	
Bachelor’s degree	4,846	40.50	41.56	41.63	
Pre-pregnancy insured status					
Insured	10,666	89.14	89.25	35.63	**<0.001**
Not insured	1,300	10.86	10.75	25.88	
Rural residence					
Urban	10,287	85.97	88.04	36.69	**<0.001**
Rural	1,679	14.03	11.96	19.08	
^a^Stressful life events (12 months)					
Family member very sick–Yes	2,384	19.92	20.76	35.53	0.454
No				34.33	
Divorced partner–Yes	660	5.52	5.31	34.40	0.946
No				34.59	
Moved–Yes	3,708	30.99	29.51	36.08	0.123
No				33.95	
Homeless–Yes	301	2.52	2.29	41.09	0.133
No				34.43	
Partner lost job–Yes	1,332	11.13	11.01	33.45	0.529
No				34.72	
Lost my job–Yes	1,562	13.05	13.11	36.44	0.261
No				34.30	
Partner/self reduced work/pay–Yes	2,734	22.85	23.41	34.77	0.866
No				34.52	
Apart from partner due to work travel–Yes	419	3.50	2.92	29.75	0.154
No				34.73	
Argued with partner more than usual–Yes	2,044	17.08	16.16	33.97	0.669
No				34.70	
Partner did not want me to be pregnant–Yes	593	4.96	4.57	31.21	0.231
No				34.74	
Problems paying rent/mortgage/bills–Yes	1,700	14.21	12.87	30.12	**0.006**
No				35.24	
Partner/self went to jail–Yes	255	2.13	1.48	17.30	**<0.001**
No				34.84	
Someone very lose – drinking/drug problem–Yes	1,162	9.71	8.97	32.50	0.3018
No				34.79	
Someone very close – died–Yes	2,213	18.49	18.08	35.16	0.671
No				34.45	
Year of new baby’s birth					
2020	6,339	52.98	60.46	35.68	**0.019**
2021	5,627	47.02	39.54	32.91	

Bold *p*-values indicate statistical significance

*PNC* prenatal care, *NH* non-Hispanic; NH other/multiracial includes American Indian or Alaska Native, Native Hawaiian or Pacific Islander, multiracial, and self-identified other racial groups; ^a^Stressful Life Events percentages that responded yes

In Table 2, we present the results from a multivariable logistic regression of virtual prenatal care. Among SDoH, adjusted odds of virtual prenatal care were higher for Hispanic (aOR = 1.61; 95% CI[1.37, 1.88]) and Asian (aOR = 1.23; 95% CI[1.01, 1.51]) than non-Hispanic white women, and notably lower for rural than urban residents (aOR = 0.51; 95% CI[0.41, 0.63]). Adjusted odds of receiving virtual prenatal care were lower for women with lower levels of educational attainment, or who had no insurance the month before becoming pregnant (aOR = 0.69; 95% CI[0.56, 0.85]). Adjusted odds were higher with increasing age and lower for those with previous births or births in 2021. Among SLEs, women who experienced homelessness (aOR = 1.90; 95% CI[1.27, 2.84]) or job loss (aOR = 1.25; 95% CI[1.04, 1.51]) had higher adjusted odds of receiving any virtual prenatal care compared to those who had not. Women who were exposed to incarceration had lower adjusted odds (aOR = 0.49; 95% CI[0.30, 0.79]) than those who were not exposed.

In a sensitivity analysis replacing the insured/uninsured indicator with a three-category measure of pre-pregnancy insurance type (private[reference], public/state, uninsured), insurance type was jointly significant (F[2,11528] = 8.06, *p*<.001): pre-pregnancy public/state insurance coverage was associated with higher adjusted odds of virtual prenatal care than private coverage (aOR = 1.27; 95% CI[1.07, 1.49]), while the uninsured-private difference was non-significant (aOR = 0.82; 95% CI[0.66, 1.03]). All other associations were substantively unchanged.

**Table 2:** Multivariable logistic regression of virtual prenatal care (*N*=11,966) – 2020−2021 Births

	aOR	SE	*p*	[Conf	Int.]
Age	1.04	0.01	<0.001	1.03	1.05
Race/ethnicity					
NH White	REF	–	–	–	–
NH Asian	1.23	0.13	**0.042**	1.01	1.51
NH Black	0.91	0.09	0.311	0.76	1.09
NH other/multiracial	0.94	0.14	0.675	0.71	1.25
Hispanic	1.61	0.13	**<0.001**	1.37	1.88
Plurality					
Multiples	REF	–	–	–	–
Singleton	1.05	0.21	0.807	0.71	1.56
Previous live births					
0	REF	–	–	–	–
1	0.78	0.05	**<0.001**	0.68	0.89
2	0.68	0.06	**<0.001**	0.57	0.82
3+	0.61	0.07	**<0.001**	0.49	0.76
Marital status					
Not married	REF	–	–	–	–
Married	1.01	0.08	0.876	0.87	1.17
Education					
No high school diploma	0.75	0.09	**0.018**	0.58	0.95
High school diploma	0.69	0.06	**<0.001**	0.58	0.83
Some college, no degree/assoc. degree	0.75	0.06	**<0.001**	0.64	0.88
Bachelor’s degree	REF	–	–	–	–
Pre-pregnancy insured status					
Insured	REF	–	–	–	–
Not insured	0.69	0.07	**<0.001**	0.56	0.85
Rural residence					
Urban	REF	–	–	–	–
Rural	0.51	0.06	**<0.001**	0.41	0.63
Stressful life events (12 months before birth)					
No	^a^REF	–	–	–	–
Family member very sick	1.03	0.08	0.690	0.89	1.20
Divorced partner	1.15	0.17	0.351	0.86	1.53
Moved	1.09	0.07	0.198	0.96	1.23
Homeless	1.90	0.39	**0.002**	1.27	2.84
Partner lost job	0.97	0.10	0.737	0.79	1.18
Lost my job	1.25	0.12	**0.017**	1.04	1.51
Partner/self reduced work/pay	1.01	0.08	0.897	0.87	1.17
Apart from partner due to work travel	0.85	0.14	0.307	0.61	1.17
Argued with partner more than usual	1.02	0.09	0.775	0.87	1.21
Partner did not want me to be pregnant	0.92	0.13	0.545	0.69	1.21
Problems paying rent/mortgage/bills	0.86	0.08	0.132	0.71	1.05
Partner/self went to jail	0.49	0.12	**0.003**	0.30	0.79
Someone very close – drinking or drug problem	0.99	0.11	0.924	0.80	1.22
Someone very close – died	1.10	0.09	0.235	0.94	1.29
Year of new baby’s birth					
2020	REF	–	–	–	–
2021	0.77	0.04	**<0.001**	0.69	0.86
Constant	0.19	0.07	**<0.001**	0.09	0.39

Bold *p*-values indicate statistical significance

*aOR* adjusted odds ratio, *SE* standard error, *p* p-value, *conf. inter.* 95% confidence interval, *NH* non-Hispanic; NH other/multiracial includes American Indian or Alaska Native, Native Hawaiian or Pacific Islander, multiracial, and self-identified other racial groups; ^a^Reference category for all stressful life events

**Fig. 2:**
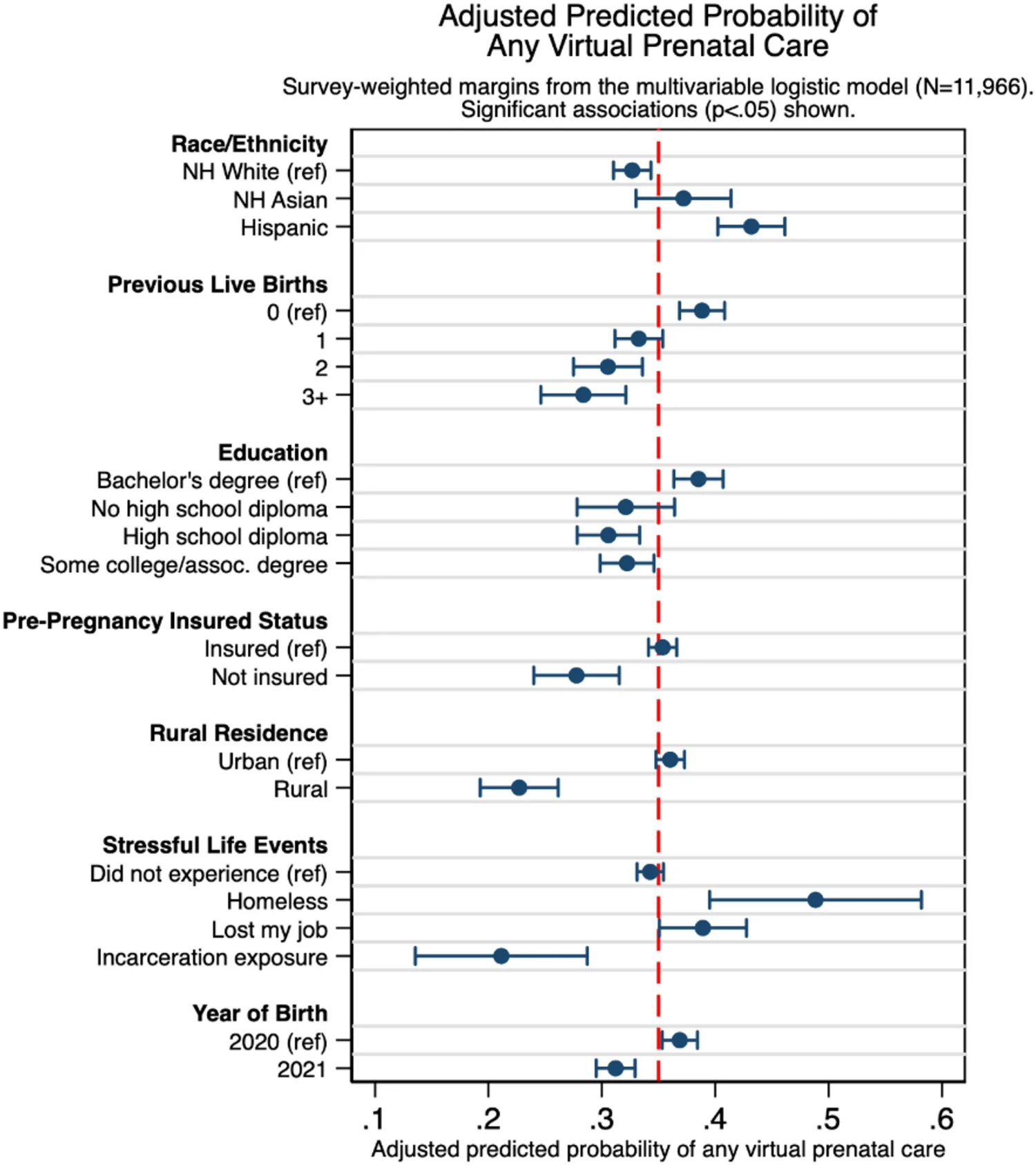
Reference categories and significantly different categories (*p*<.05) are shown; non-significant categories are omitted from the figure but retained in the model, which also adjusted for age, plurality, marital status, and additional stressful life events. The dashed line marks the sample mean (0.35)

Figure 2 presents adjusted predicted probabilities of any virtual care for each independent variable. The absolute differences were most pronounced for rural residence (0.23 vs. 0.36 for urban) and incarceration exposure (0.21 vs. 0.35) and were notable for homelessness (0.49 vs. 0.34) and Hispanic ethnicity (0.43 vs. 0.33 for non-Hispanic white women).

## Discussion

This study examined the utilization of virtual prenatal care and its associations with SDoH and SLEs. Just over a third (35%) of women used virtual prenatal care, which is consistent with other estimates around this time period (Gao et al., 2021; Gourevitch et al., 2023; Lee & Manalew, 2023) but higher than estimates for infants born in November 2020 (17%) (Acharya et al., 2023). Our analyses revealed *greater* odds of virtual prenatal care for older (vs. younger), Asian and Hispanic women (vs. white women) and *lower* odds for women who had previously given birth (vs. no prior live births), had lower levels of education (vs. higher education), lacked insurance in the months prior to pregnancy (vs. insured), and lived in rural (vs. urban) counties. Our analyses also revealed that some SLEs were associated with lower odds (incarceration exposure) of virtual prenatal care, while others SLEs were associated with greater odds (homelessness and job loss). Together, our findings suggest that SDoH—both structural stratifiers and intermediary determinants—are important considerations for understanding virtual prenatal care utilization (Solar & Irwin, 2010).

Consistent with our findings, prior research revealed younger age and lower education to be associated with less virtual prenatal care utilization (Gao et al., 2021; Gourevitch et al., 2023; Lee & Manalew, 2023). Some evidence suggests the association between less education and lower virtual prenatal care utilization may be due to a lack of private space and/or technological barriers (Lee & Manalew, 2023). Our findings confirm prior research which demonstrated less virtual prenatal care utilization among uninsured women (Gourevitch et al., 2023). We also find, along with two other studies, evidence of disparities in virtual prenatal care utilization across urban/rural residences (Gao et al., 2021; Gourevitch et al., 2023; Hung et al., 2024). However, while our finding of lower odds of utilization among rural residents is consistent with other analyses utilizing PRAMS data (Gourevitch et al., 2023), it runs counter to another study that showed higher utilization among rural residents (Gao et al., 2021). Although one single-site study found minoritized racial groups were less likely than white patients to utilize virtual prenatal care (Gao et al., 2021) our findings align with several nationally-focused studies which found Hispanic and Asian patients are more likely to utilize virtual prenatal care (Gourevitch et al., 2023; Hung et al., 2024; Lee & Manalew, 2023). Although the reasons for these differences are still unclear, some evidence indicates a lower preference for in-person care among Hispanic individuals (Lee & Manalew, 2023).

Three SLEs were significant predictors for virtual prenatal care; however, the associations were not always as expected. Women who reported experiencing homelessness or job loss in the year prior to their pregnancy had greater odds of receiving virtual prenatal care. Increased odds of utilization for women experiencing these SLEs was an unexpected finding and warrants further empirical investigation. Some research suggests virtual care can help overcome transportation barriers (Oluyede et al., 2022), which is often a challenge for those experiencing homelessness or job loss (Murphy, 2019). Thus, homelessness and job loss may be linked to increased odds of virtual prenatal care utilization out of necessity due to increased barriers to in-person care (e.g., transportation). Incarceration exposure was associated with significantly lower odds of receiving virtual prenatal care. Like many social stressors, exposure to incarceration may proliferate into additional stressors, and has been linked to several barriers to in-person prenatal care (e.g., lack of transportation, lack of child care, busy schedules) (Testa & Jackson, 2020). Future investigations should consider the link between criminal justice contact and “systems avoidance,” the tendency to avoid surveilling institutions including medical care (Brayne, 2014).

Our findings confirm prior research demonstrating lower odds of receiving any virtual prenatal care among women who gave birth in 2021 versus 2020 (Lee & Manalew, 2023). These results align with previous research findings that, across many healthcare specialties and patient payer types, telehealth utilization peaked in the early months of the COVID-19 PHE and declined during mid- to-late 2020 but remained well above pre-PHE utilization rates (Mulvaney-Day et al., 2022).

### Limitations

This study has limitations that should be considered and could be improved in future research. First, although we had data across multiple years, we did not have multiple data points for any individual in this study. We were only able to assess associations and could not determine causality. Second, due to small subsamples, we aggregated racial/ethnic groups who may vary significantly in their healthcare-seeking and utilization behaviors, and more granular levels of ethnicity were not available. Third, there are likely relevant variables we could not account for in this study, such as the timing of stressful life events relative to the pregnancy, length of sentence during incarceration exposure, prior telehealth utilization, digital health literacy, and state-level telehealth policies. Fourth, the wording of the virtual prenatal care item on the PRAMS survey did not allow for an examination of disparities in types of virtual prenatal care, namely between audio-only and audio-video visits. Fifth, the incarceration exposure item does not allow us to determine if the respondent or their spouse was incarcerated. Finally, PRAMS survey data yield estimates representative of live births within participating jurisdictions but may not be representative of all births in the U.S.

## Implications for Public Health

In this multistate study of prenatal care utilization, we found that approximately a third of women utilized virtual rather than in-person only delivery of prenatal care. Several SDoH were associated with virtual prenatal care utilization, including race/ethnicity, education, insured status, and rural residence. We did not find substantial negative impacts of SLEs in the last 12 months on virtual prenatal care utilization. However, some SLEs, such as homelessness and job loss, significantly increased the odds of virtual prenatal care utilization, while the SLE of incarceration exposure significantly decreased the odds of utilization.

These results add to a growing body of literature indicating that virtual prenatal care utilization is not straightforwardly related to indicators of SDoH or SLEs. Instead, these results suggest that the likelihood of virtual prenatal care utilization may arise from a position of advantage or protection from stress in some circumstances and disadvantage or stressful life events in others. For example, experiences such as job loss or homelessness may make virtual prenatal care the only option for those without transportation or money for fuel. In contrast, less education or greater incarceration exposure may bring with them additional challenges to utilizing virtual care. Attention to the role SDoH may play in shaping virtual prenatal care utilization will be critical to how this increasingly common mode of care impacts health outcomes further downstream.

## Data Availability

PRAMS Data and documentation are publicly available from the Centers for Disease Control.

## References

[CR1] Acharya, M., Ali, M. M., Hayes, C. J., Bogulski, C. A., Magann, E. F., & Eswaran, H. (2023). Trends in telehealth visits during pregnancy, 2018 to 2021. *JAMA Network Open,**6*(4), Article e236630. 10.1001/jamanetworkopen.2023.6630 37014645 10.1001/jamanetworkopen.2023.6630 PMC10074218

[CR2] Blakeney, E. L., Herting, J. R., Bekemeier, B., & Zierler, B. K. (2019). Social determinants of health and disparities in prenatal care utilization during the Great Recession period 2005–2010. *BMC Pregnancy and Childbirth,**19*(1), Article 390. 10.1186/s12884-019-2486-1 31664939 10.1186/s12884-019-2486-1 PMC6819461

[CR3] Braveman, P., Marchi, K., Egerter, S., Pearl, M., & Neuhaus, J. (2000). Barriers to timely prenatal care among women with insurance: The importance of prepregnancy factors. *Obstetrics & Gynecology*, *95*(6, Part 1), 874–880. 10.1016/S0029-7844(00)00780-8 10831984 10.1016/s0029-7844(00)00780-8

[CR4] Brayne, S. (2014). Surveillance and system avoidance: Criminal justice contact and institutional attachment. *American Sociological Review,**79*(3), 367–391. 10.1177/0003122414530398

[CR5] Cohen, S., Murphy, M. L. M., & Prather, A. A. (2019). Ten surprising facts about stressful life events and disease risk. *Annual Review of Psychology,**70*(1), 577–597. 10.1146/annurev-psych-010418-102857 29949726 10.1146/annurev-psych-010418-102857 PMC6996482

[CR6] Dumont, D. M., Wildeman, C., Lee, H., Gjelsvik, A., Valera, P., & Clarke, J. G. (2014). Incarceration, maternal hardship, and perinatal health behaviors. *Maternal and Child Health Journal*, *18*(9), 2179–2187. 10.1007/s10995-014-1466-3 24615355 10.1007/s10995-014-1466-3 PMC4161663

[CR7] Gadson, A., Akpovi, E., & Mehta, P. K. (2017). Exploring the social determinants of racial/ethnic disparities in prenatal care utilization and maternal outcome. *Seminars in Perinatology, Strategies to Reduce Racial/Ethnic Disparities in Maternal Morbidity and Mortality,**41*(5), 308–317. 10.1053/j.semperi.2017.04.008 10.1053/j.semperi.2017.04.008 28625554

[CR8] Gao, C., Osmundson, S., Malin, B., & Chen, Y. (2021). Prenatal Telehealth During the Pandemic: Sociodemographic and Clinical Associations. *Telehealth and Medicine Today*. 10.30953/tmt.v6.279

[CR9] Gourevitch, R. A., Anyoha, A., Ali, M. M., & Novak, P. (2023). Use of prenatal telehealth in the first year of the COVID-19 pandemic. *JAMA Network Open,**6*(10), Article e2337978. 10.1001/jamanetworkopen.2023.37978 37815833 10.1001/jamanetworkopen.2023.37978 PMC10565607

[CR10] Heaman, M. I., Martens, P. J., Brownell, M. D., Chartier, M. J., Derksen, S. A., & Helewa, M. E. (2019). The association of inadequate and intensive prenatal care with maternal, fetal, and infant outcomes: A population-based study in Manitoba, Canada. *Journal of Obstetrics and Gynaecology Canada,**41*(7), 947–959. 10.1016/j.jogc.2018.09.006 30639165 10.1016/j.jogc.2018.09.006

[CR11] Hung, P., Yu, J., Harrison, S. E., Liu, J., Promiti, A., Odahowski, C., Campbell, B. A., Chatterjee, A., Boghossian, N. S., Cai, B., Liang, C., Li, J., Li, X., National COVID Cohort Collaborative Consortium. (2024). Racial and Ethnic and Rural Variations in the Use of Hybrid Prenatal Care in the US. *JAMA Network Open,**7*(12), e2449243. 10.1001/jamanetworkopen.2024.49243 39641928 10.1001/jamanetworkopen.2024.49243 PMC11624583

[CR12] Ingram, D. D., & Franco, S. J. (2014). 2013 NCHS urban–rural classification scheme for counties (Data Evaluation and Methods Research No. 166; Vital and Health Statistics 2). National Center for Health Statistics. 24776070

[CR13] Lee, J., & Manalew, W. S. (2023). Reasons for Not Pursuing Virtual Prenatal Care in 2020 Through 2021 and Policy Implications. *Telemedicine and E-Health*, *29*(10), 1492–1503. 10.1089/tmj.2022.0492 36787485 10.1089/tmj.2022.0492 PMC10589501

[CR14] Mezuk, B., Rafferty, J. A., Kershaw, K. N., Hudson, D., Abdou, C. M., Lee, H., Eaton, W. W., & Jackson, J. S. (2010). Reconsidering the Role of Social Disadvantage in Physical and Mental Health: Stressful Life Events, Health Behaviors, Race, and Depression. *American Journal of Epidemiology*, *172*(11), 1238–1249. 10.1093/aje/kwq283 20884682 10.1093/aje/kwq283 PMC3025628

[CR15] Mulvaney-Day, N., Dean, D., Miller, K., & Camacho-Cook, J. (2022). Trends in Use of Telehealth for Behavioral Health Care During the COVID-19 Pandemic: Considerations for Payers and Employers. *American Journal of Health Promotion*, *36*(7), 1237–1241. 10.1177/08901171221112488e 36003014 10.1177/08901171221112488e PMC9412131

[CR16] Murphy, E. R. (2019). Transportation and homelessness: A systematic review. *Journal of Social Distress and the Homeless*, *28*(2), 96–105. 10.1080/10530789.2019.1582202

[CR17] Nelson, D. B., Moniz, M. H., & Davis, M. M. (2018). Population-level factors associated with maternal mortality in the United States, 1997–2012. *BMC Public Health,**18*(1), Article 1007. 10.1186/s12889-018-5935-2 30103716 10.1186/s12889-018-5935-2 PMC6090644

[CR18] Oluyede, L., Cochran, A. L., Wolfe, M., Prunkl, L., & McDonald, N. (2022). Addressing transportation barriers to health care during the COVID-19 pandemic: Perspectives of care coordinators. *Transportation Research Part A Policy and Practice*, *159*, 157–168. 10.1016/j.tra.2022.03.010 35283561 10.1016/j.tra.2022.03.010 PMC8898700

[CR19] Radke, S. M., Smeins, L., Ryckman, K. K., & Gruca, T. S. (2023). Closure of labor & delivery units in rural counties is associated with reduced adequacy of prenatal care, even when prenatal care remains available. *The Journal of Rural Health,**39*(4), 746–755. 10.1111/jrh.12758 36999217 10.1111/jrh.12758

[CR20] Shulman, H. B., D’Angelo, D. V., Harrison, L., Smith, R. A., & Warner, L. (2018). The Pregnancy Risk Assessment Monitoring System (PRAMS): Overview of design and methodology. *American Journal of Public Health,**108*(10), 1305–1313. 10.2105/AJPH.2018.304563 30138070 10.2105/AJPH.2018.304563 PMC6137777

[CR21] Simmons, E., & Austin, A. E. (2022). Association of prenatal substance use with prenatal and postpartum care: Evidence from the Pregnancy Risk Assessment Monitoring System, 2016–2019. *Preventive Medicine,**159*, Article 107065. 10.1016/j.ypmed.2022.107065 35461958 10.1016/j.ypmed.2022.107065 PMC10018998

[CR22] Slaughter-Acey, J. C., Caldwell, C. H., & Misra, D. P. (2013). The Influence of Personal and Group Racism on Entry into Prenatal Care among African American Women. *Women’s Health Issues: Official Publication of the Jacobs Institute of Women’s Health*. 10.1016/j.whi.2013.08.001 24041828 10.1016/j.whi.2013.08.001 PMC3845454

[CR23] Solar, O., & Irwin, A. (2010). A conceptual framework for action on the social determinants of health. World Health Organization, Discussion Paper Series on Social Determinants of Health, 2, 76.

[CR24] Testa, A., & Jackson, D. B. (2020). Incarceration Exposure and Barriers to Prenatal Care in the United States: Findings from the Pregnancy Risk Assessment Monitoring System. *International Journal of Environmental Research and Public Health*. 10.3390/ijerph17197331 33049968 10.3390/ijerph17197331 PMC7578954

[CR25] Testa, A., Jackson, D. B., Vaughn, M. G., & Bello, J. K. (2020). Incarceration as a unique social stressor during pregnancy: Implications for maternal and newborn health. *Social Science & Medicine,**246*, Article 112777. 10.1016/j.socscimed.2019.112777 31918349 10.1016/j.socscimed.2019.112777

[CR26] Testa, A., Lee, J., Semenza, D. C., Jackson, D. B., Ganson, K. T., & Nagata, J. M. (2023). Intimate partner violence and barriers to prenatal care. *Social Science & Medicine,**320*, Article 115700. 10.1016/j.socscimed.2023.115700 36708607 10.1016/j.socscimed.2023.115700

[CR27] Thurston, H., Fields, B. E., & White, J. (2021). Does increasing access to prenatal care reduce racial disparities in birth outcomes? *Journal of Pediatric Nursing,**59*, 96–102. 10.1016/j.pedn.2021.01.012 33588292 10.1016/j.pedn.2021.01.012

[CR28] Yan, J. (2017). The Effects of Prenatal Care Utilization on Maternal Health and Health Behaviors. *Health Economics*, *26*(8), 1001–1018. 10.1002/hec.3380 27374163 10.1002/hec.3380

